# Green Processing of *Ilex guayusa*: Antioxidant Concentration and Caffeine Reduction Using Encapsulation by Supercritical Antisolvent Process

**DOI:** 10.3390/molecules29225309

**Published:** 2024-11-11

**Authors:** Miguel Ángel Meneses, Jhulissa Guzmán, Jhulissa Cabrera, Jorge Magallanes, Eduardo Valarezo, María del Cisne Guamán-Balcázar

**Affiliations:** 1 Departamento de Química, Universidad Técnica Particular de Loja, Loja 1101608, Ecuador; bevalarezo@utpl.edu.ec (E.V.); mcguaman@utpl.edu.ec (M.d.C.G.-B.); 2 Carrera de Bioquímica y Farmacia, Universidad Técnica Particular de Loja, Loja 1101608, Ecuador; jmguzman11@utpl.edu.ec; 3 Carrera de Ingeniería Química, Universidad Técnica Particular de Loja, Loja 1101608, Ecuador; jacabrera25@utpl.edu.ec; 4 Master in Applied Chemistry Program, Universidad Técnica Particular de Loja, Loja 1101608, Ecuador; jmagallanes5346@upse.edu.ec; 5 Carrera de Biología, Universidad Estatal Península de Santa Elena, Santa Elena 240201, Ecuador; 6 Carrera de Ecología y Conservación Ambiental, Universidad Estatal Península de Santa Elena, Santa Elena 240201, Ecuador

**Keywords:** supercritical antisolvent extraction (SAE), *Ilex guayusa*, Polyvinylpyrrolidone (PVP), chlorogenic acid, decaffeination, encapsulation

## Abstract

This study investigated the valorization of *Ilex guayusa* leaves by producing a low-caffeine, antioxidant-rich product through the supercritical antisolvent extraction (SAE) process. The objective was to concentrate the antioxidants while selectively reducing the caffeine. The SAE treatments were conducted using an ethanolic extract of guayusa leaves under varying pressure (80 bar–150 bar) and temperature (35–45 °C) conditions to improve the recovery of chlorogenic acid (CGA) and caffeine fractionation. The co-precipitation of antioxidants with polyvinylpyrrolidone (PVP) (ratio 1:1–1:2 mass/mass) as an encapsulant was also studied. The SAE precipitates were analyzed for their recovery yield, CGA and caffeine contents, antioxidant activity, and total phenols. Based on the statistical analysis, the optimal conditions for the SAE were 120 bar and 45 °C. Under these conditions, the CGA concentration increased from 43.02 mg/g extract to 237 mg/g precipitate, while the caffeine was reduced to less than 1% mass. Co-precipitation with PVP improved the recovery yield by more than two times than the SAE alone while maintaining the caffeine content below 1% mass. Additionally, the co-precipitation with PVP facilitated the formation of spherical microparticles, indicating successful encapsulation of the bioactive compounds, with an IC_50_ of 0.51 ± 0.01 mg/mL for DPPH and 0.18 ± 0.01 mg/mL for ABTS. These results highlight the effectiveness of the SAE co-precipitation process in developing low-caffeine functional ingredients with potential food and pharmaceutical applications.

## 1. Introduction

*Ilex guayusa* (Guayusa) is a plant native to the Amazon that belongs to the Aquifoliaceae family, and it is consumed in beverages by the indigenous population of Ecuador, Bolivia, Peru, and Colombia. This species grows between 200 and 2000 m.a.s.l. In Ecuador, it is cultivated in the Amazonian provinces of Sucumbíos, Napo, Pastaza, Morona Santiago, and Zamora Chinchipe [1]. Traditionally, guayusa has been used by the indigenous people of the Amazon region in rituals and ceremonies and “as a source of energy”, as well as to treat various ailments and promote general health [2].

According to Arteaga-Crespo et al. [3], the traditional uses of *Ilex guayusa* tea are as a stimulant, diuretic, and stomach tonic, in the same way a tea from the leaves is reported to have beneficial results against diabetes, venereal diseases, flu, and body aches; in addition, emphasis is placed on the use to increase fertility and libido.

During the last decade, several studies were carried out regarding the phytochemical composition of *Ilex guayusa* leaves, reporting an interesting source of secondary metabolites, such as pentacyclic triterpenoid derivatives, xhanthines, flavonoids, saponins, and chlorogenic acid derivatives [4,5,6].

In reference to [6], the following secondary metabolites from *Ilex guayusa* leaves have been reported: Caffeine 36 mg/mL, Theobromine 0.3 mg/mL, Chlorogenic acids 52 mg/mL, total polyphenols 10 mg/mL, Catechin 2 mg/mL, Isoflavones 0.8 mg/mL, Epicatechin 0.179 mg/mL, Epicatechin gallate (ECG), 0.199 mg/mL, Epigallocatechin gallate (EGCG) 0.0876 mg/mL, Epigallocatechin (EGC) 1.11 mg/mL, Kaempferol trace, and Naringin trace.

Caffeine is a methylxanthine (1,3,7-trimehylxanthine) well known for its stimulating effects on the central nervous system, and it is widely found in plants, such as coffee, tea, and guayusa. Caffeine is one of the most widely consumed substances in the world as natural and processed products. It has been studied for many years due to its favorable effects in humans, such as ergogenic and antioxidant properties. However, its consumption has also been linked to adverse effects, such as reduced tonus of the lower esophageal sphincter, overstimulation of the central nervous system, and the potential for dependence [7]. Given these concerns, there is growing interest in decaffeinated products that retain the health benefits associated with other bioactive compounds, particularly antioxidants, without the negative effects of caffeine, such as anxiety, nervousness, and hypertension.

Chlorogenic acids (CGAs) are phenolic compounds from which 5-*O*-Caffeoylquinic acid is the most abundant in guayusa leaves [6]. CGA presents beneficial effects, such as antioxidant, anti-inflammatory, and neuroprotective effects [8]. These compounds have also been associated with improving cardiovascular health, reducing the risk of type 2 diabetes, and mitigating inflammation-related conditions [9]. The synergistic interactions between caffeine and CGA, such as those naturally present in guayusa, were shown to enhance cognitive function and mood [8].

In the food, chemical, and pharmaceutical industries, there is a significant interest in natural compounds for developing functional foods or as alternatives to synthetic antioxidants, such as butylhydroxytoluene (BHT) and butylhydroxyanisole (BHA), which are commonly used in the food preservation industry [9]. Secondary metabolites, such as caffeine and chlorogenic acids (CGAs), are extracted using traditional methods, such as soxhlet extraction or maceration. However, these techniques present notable drawbacks, including the use of toxic solvents, high temperatures, prolonged extraction times, and the need for additional purification steps for recovering both the extract and the solvent [10]. Supercritical fluid extraction (SFE) not only allows for overcoming these limitations but also aligns with environmentally sustainable practices by enabling efficient and selective extraction by fine-tuning the temperature and pressure. Carbon dioxide (CO_2_) is the most widely used supercritical solvent due to its non-toxic, non-flammable, and non-corrosive nature; its ability to be recycled and reused in a closed-loop process; and its easily-accessible critical point (T_C_ = 31.13 °C, P_C_ = 7.38 MPa), making it an ideal candidate for industrial applications. Furthermore, the use of CO_2_ sourced from industrial waste streams contributes to a reduction in overall carbon emissions, reinforcing its role in sustainable processing. Supercritical CO_2_ extraction is a green alternative that is particularly advantageous for preserving the integrity of bioactive compounds during extraction, which is crucial in producing high-quality products for functional foods and pharmaceuticals.

The supercritical antisolvent (SAS) technique has gained attention for the separation and encapsulation of polar bioactive molecules [11,12]. In the SAS technique, supercritical CO_2_ acts as an antisolvent by precipitating nonsoluble solutes from a polar organic solution. This method has been effectively used in food processing to isolate bioactive components while maintaining their chemical integrity and biological activity [13]; it is named supercritical antisolvent extraction (SAE). Villanueva et al. [14] reported on the application of SAE for producing a high-catechins/low-caffeine powder from tea leaves, where they reported the selective precipitation of catechins versus caffeine. Quintana et al. [15] performed the simultaneous fractionation and precipitation of antioxidants from an ethanolic rosemary extract, where they obtained a precipitate enriched in rosmarinic acid, while carnosinic acid and carnosol were concentrated in the oleoresin fraction. Additionally, SAE is versatile for co-precipitating bioactive compounds with protective polymers, creating encapsulated products with enhanced stability and bioavailability. For instance, SAE was applied to encapsulate antioxidants from mango leaves [16] and moringa leaves [17]. The SAE processing was also applied to a lycopene extract and its co-precipitation was performed with PVP [18].

Polyvinylpyrrolidone (PVP) is a synthetic, water-soluble biopolymer commonly used as an encapsulating agent due to its ability to improve the stability, bioaccessibility, and bioavailability of bioactive compounds [19]. PVP is approved by the U.S. Food and Drug Administration (FDA) as an inactive ingredient in various pharmaceutical formulations, making it a safe and effective choice for food and drug applications [20]. The SAS processing of PVP can form microparticles or, depending on supercritical conditions and solvent selection, other morphologies, such as nanoparticles and sub-microparticles [21,22]. This adaptability makes PVP ideal for encapsulating sensitive compounds, like antioxidants, ensuring their functional properties are preserved throughout processing and storage.

The primary objective of this study was to valorize the antioxidant properties of *Ilex guayusa* leaves by developing a low-caffeine, antioxidant-rich product encapsulated with polyvinylpyrrolidone (PVP) through a supercritical antisolvent extraction (SAE) process. This research focused on selectively reducing the caffeine content while preserving and enhancing the antioxidant components, such as chlorogenic acids, present in the guayusa extract. The encapsulation using PVP aimed to stabilize and protect these antioxidant compounds, improving their bioavailability and potential health benefits. In this study, the antioxidant capacities and chlorogenic acid and caffeine contents of both the encapsulated and fractionated products were evaluated, in addition to the particle size distributions of the encapsulated samples. The SAE process enabled efficient fractionation and co-precipitation of the extract and PVP and produced high-quality encapsulated particles with controlled release properties, potentially offering a functional food ingredient or supplement.

## 2. Results

### 2.1. Characterization of the Ethanolic Extract

The total ethanolic extract was characterized (Table 1) by the extraction yield, total phenol content, DPPH (2,2-diphenyl-1-picrylhydrazyl) and ABTS (2,2′-azino-bis(3-ethylbenzothiazoline-6-sulfonic acid)) antioxidant capacities, caffeine concentration, and phenolic composition. These results were the basis for understanding the effects of supercritical precipitation as a technique to produce an enriched antioxidant product when comparing both results. The total extract obtained with absolute ethanol allowed for using SC-CO_2_ as an antisolvent to avoid operational issues in the equipment, such as freezing of the solvent in the depressurize steps and to ensure that the ethanol–extract–CO_2_ mixture was under moderately supercritical conditions. The concentrations of the caffeine and chlorogenic acid based on the plant material dry weight (DW) were 26 mg/g DW and 14.2 mg/g DW, respectively. Chlorogenic acid and caffeine were also analyzed in the following SAE fraction investigations.

### 2.2. Supercritical Antisolvent Extraction of Antioxidants and Caffeine

#### 2.2.1. SAE Processing

The supercritical antisolvent extraction allowed for obtaining two fractions. The precipitate product, in the solid state, corresponded to the microparticles collected in the precipitation vessel due to the supersaturation caused by the effect of supercritical CO_2_. The fractionate product, in the liquid state, corresponded to the solubilized compounds in the supercritical mixture of CO_2_–ethanol and condensed in a separator vessel.

The recovery yield of each of the nine treatments is presented in Table 2 for both fractions; for the fractionated product, the liquid ethanol was eliminated by evaporation. The recovery yield in the precipitate products was between 7.06% and 24.54%, while for the fractionated product, it was between 34.24% and 73.27%.

The statistical analysis showed that under the experimental conditions, the pressure and the pressure–temperature interaction had effects on the precipitation yield (*p* < 0.05). From the interaction graph, a pressure of 120 bar and temperature of 35 °C was determined as the conditions with the higher recovery yield in the precipitated fraction (24.54 ± 0.40%). At 120 bar, the increase in temperature produced a diminishment of the recovery yield.

#### 2.2.2. Total Phenol Content

All the extracts produced in the supercritical antisolvent treatments were analyzed by the total phenols content (TPC). The results (Table 3) show values from 417.08 to 561.52 mg GAE/g extract in the precipitated fraction and 8 to 29.90 mg GAE/g extract in the fractionated fraction. The statistical analysis showed that only the temperature had an effect on the TPC (*p* < 0.05); the temperature of 45 °C had the highest results (*p* < 0.05) for all the pressures evaluated. The precipitated fraction with the higher value of TPC (561.52 ± 15.71 mg GAE/g extract) corresponded to 120 bar and 45 °C (treatment 9).

#### 2.2.3. Antioxidant Capacity

The antioxidant capacity of the two fractions of the supercritical antisolvent was evaluated by ABTS and DPPH methods. The results obtained are shown in Table 4. For the ABTS results, the values were between 185.95 and 218.63 µMol TE/g extract in the precipitated fraction and between 21.19 and 56.87 µMol TE/g extract in the fractionated fraction, while for the DPPH results, the values were between 601.33 and 849.36 µMol Trolox/g extract in the precipitated fraction and between 19.83 and 41.10 µMol TE/g extract in the fractionated fraction. In all treatments, the antioxidant activity of the precipitated product was considerable higher than the fractionated product. The statistical analysis showed no effect of pressure and temperature (*p* > 0.05) on the ABTS and DPPH antioxidant results for each fraction. As a rule, the precipitated fraction showed a higher antioxidant capacity than the fractionated one.

#### 2.2.4. Chemical Composition of Guayusa Extract

The chemical analysis of the phenolics compounds, carried out by HPLC, allowed for correctly identifying two compounds, chlorogenic acid and caffeine, when compared with the retention time of the HPLC grade standards. In the SAE treatments, the precipitated fraction was the product with the higher antioxidant capacity; in this fraction, chlorogenic acid was the major compound identified, while caffeine was present in lower quantities. Table 5 shows the concentrations of these compounds for every treatment.

The statistical analysis was performed over the chlorogenic acid concentration, which was the most abundant phenolic compound identified. The ANOVA showed that the pressure and the P*T interaction had effects on the chlorogenic acid concentration. From the P*T interaction graph, a pressure of 120 bar and temperatures of 35 °C and 45 °C were determined as the conditions with the higher concentrations of chlorogenic acid in the precipitated fraction, with 237.86 ± 13.83 and 213.76 ± 22.39 mg/g precipitate, respectively. Regarding the caffeine concentrations, in the SAE precipitate, it ranged from 4.98 to 14.51 mg/g precipitate, while in the SAE fractionate, it was from 70.96 to 116.78 mg/g fractionate. It is noted that caffeine was fractionated from the antioxidant compounds.

### 2.3. Supercritical Antisolvent Co-Precipitation of Guayusa Extract

From the SAE treatments, it was possible to identify the supercritical conditions of pressure and temperature that increased the recovered product yield and the concentration of chlorogenic acid, which corresponded to 120 bar and 35 °C. Table 6 and Table 7 show the results for co-precipitation; additional treatments at 150 bar were included due to the value for the 120 bar pressure corresponding to the extreme value assayed in the SAE. Similarly, a temperature of 45 °C was assayed considering the best operative conditions for the total phenols content and chlorogenic acid concentration (120 bar and 45 °C).

Table 6 shows that the presence of polyvinylpyrrolidone (PVP) increased by more than two times the recovery yield of the co-precipitate compared with the SAE result. From the statistical analysis, only the extract–PVP ratio had a significant effect (*p* < 0.05) on the recovery yield and the content of total phenols. The use of PVP in a 1:2 ratio increased the recovery yield, while at the same time reduced the total phenols content when compared with a 1:1 ratio. The treatment performed at a pressure of 150 bar of pressure did not improve the recovery yield compared with the other treatments.

Regarding the chlorogenic acid concentration, the three factors showed statistical effects (*p* < 0.05); from the main effects graphs, the conditions that increased the concentration of chlorogenic acid were a 1:1 extract–PVP ratio, a pressure of 120 bar, and a temperature of 35 °C. The recovery yield treatments at 150 bar and 45 °C did not improve the concentrations of chlorogenic acid in the co-precipitates. For all the co-precipitate treatments, the caffeine content was less than 1% mass concentration. Fisher’s mean difference test was performed to compare the co-precipitate responses against the SAE; for all the responses, there were statistical differences due to the PVP material added for the coprecipitation.

Table 7 show the results for IC_50_ of the co-precipitates. The IC_50_ values for the SAE precipitate (treatment 1) were 0.04 ± 0.00 mg/mL for DPPH and 0.18 ± 0.01 mg/mL for ABTS; these values were lower than the co-precipitates, indicating high antioxidant activity. The IC_50_ values of the SAE co-precipitates ranged from 0.51 to 1 mg CP/mL for the DPPH assay and ranged from 0.74 to >1 mg/mL for the ABTS assay. It should be noted that a higher IC_50_ corresponds to minor antioxidant activity. Among the co-precipitates, those obtained with a 1:1 extract–PVP ratio showed the lowest IC_50_ values. Specifically, at 120 bar and 35–45 °C, the IC_50_ values were 0.51–0.54 mg CP/mL for DPPH and 0.77–0.74 mg CP/mL for ABTS.

### 2.4. Particle Size Distribution of Guayusa Co-Precipitates

**Figure 1:**
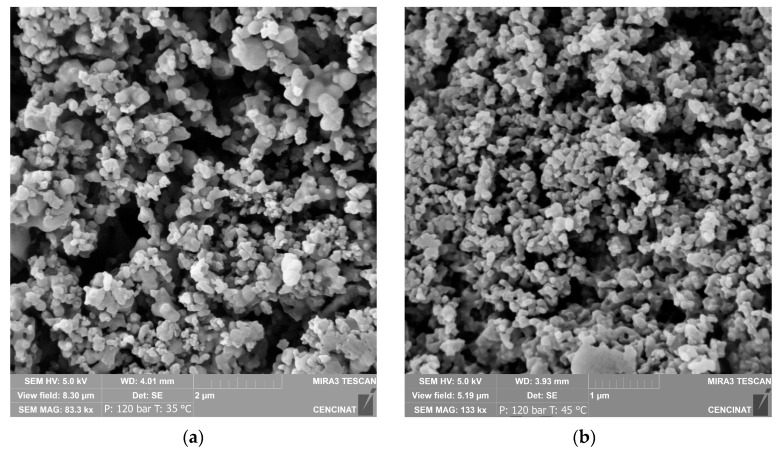
SEM images of guayusa antioxidants precipitated for treatments 1 (**a**) and 6 (**b**).

The particle size and distribution were analyzed for the treatments conducted at 120 bar and a temperature of 35 °C (SAE), and 120 bar and 45 °C (co-precipitate, extract–PVP 1:1) (Figure 1). The microparticles from the SAE precipitate showed slight agglomeration, with a mean particle size of 0.23 ± 0.07 µm. In contrast, the co-precipitate microparticles appeared as free particles with a mean size of 0.24 ± 0.08 µm. In addition, the particle size was measured for treatments 2 and 7, with values of 0.29 ± 0.11 µm and 0.19 ± 0.07 µm, respectively.

**Figure 2:**
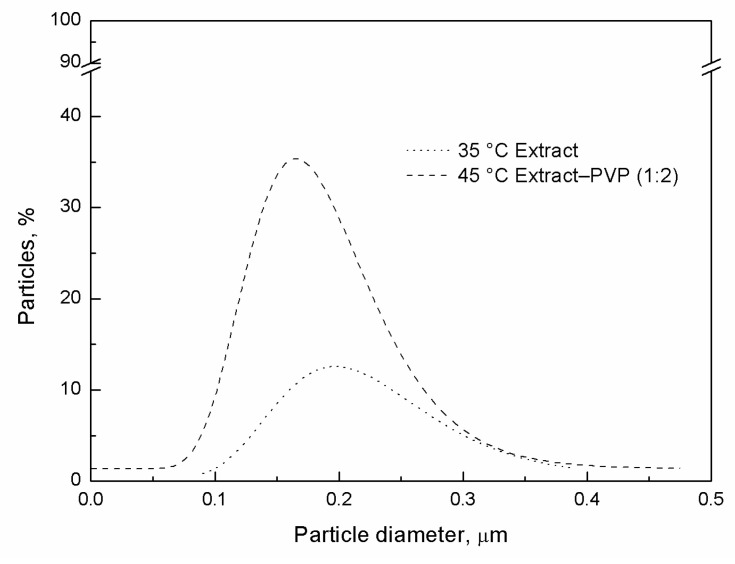
Particle size distribution is expressed as the frequency of the number of particles precipitated at 120 bar.

The presence of PVP improved the particle size distribution (PSD), as observed in Figure 2, where the co-precipitate exhibited a more homogeneous distribution compared with the SAE precipitate. Measuring the particle size and PSD is essential for ensuring the stability, bioavailability, and functionality of the final product, particularly for applications in functional foods and pharmaceuticals.

## 3. Discussion

The total extraction yield of 33.61 ± 2.27% was notably higher than the values reported by Cadena-Carrera et al. [23], who observed yields of 19.66 ± 3.91% using Soxhlet extraction with ethanol, 16.11 ± 0.64% with ethyl acetate, and 3.08 ± 0.16% with supercritical CO_2_ extraction. This increase in yield may be attributed to the choice of solvent, namely, ethanol, which is known for its efficacy in extracting polar compounds, along with the optimized extraction conditions used in this study. Factors such as solvent concentration, temperature, extraction time, and agitation are critical for improving the yield, as is the nature of the vegetable matrix and the polarity of compounds. Nevertheless, further optimization of these parameters could enhance the extraction process and reduce the co-extraction of undesirable compounds.

The TPC of the ethanolic extract was 2.63 g GAE/100 g DW, which is comparable with previously reported values. Arteaga-Crespo et al. [3] obtained a TPC of 3.46 g GAE/100 g DW with ultrasound-assisted extraction, while Villacis-Chiriboga et al. [5] reported values of 3.34 g GAE/100 g DW and 2.14 g GAE/100 g DW for adult and young leaves, respectively. Higher values observed by García-Ruiz et al. [6] (5.48 g GAE/100 g DW) and Pardau et al. [24] (5.44 and 6.72 mg GAE/g) could have been due to variations in the extraction techniques, solvent types, leaf maturity, and geographical origin.

The antioxidant activity was measured as 75.6 ± 12.1 µMol Trolox/g extract for DPPH and 59.80 ± 5.72 µMol TE/g extract for ABTS. Comparatively, Arteaga-Crespo et al. [3] reported an ABTS value of 40.71 µMol TE/g DW, while Villacis-Chiriboga et al. [5] observed DPPH values that ranged from 124.7 to 161.9 µMol Trolox/g DW. Other studies have reported higher values, such as García-Ruiz et al. [6] (330 µMol TE/g DW for DPPH) and Pardau et al. [24] (115.1 µMol TE/g DW). In contrast, supercritical CO_2_ extraction showed a lower ABTS antioxidant capacity of 1.45 µMol TE/g DW, which increased to 2.98 µMol Trolox/g DW when ethanol was used as the cosolvent [23].

The chlorogenic acid and caffeine concentrations in the extract were 43.02 ± 5.23 mg/g extract (14.45 ± 1.78 mg/g DW) and 79.16 ± 3.56 mg/g extract (26.60 ± 1.20 mg/g DW), respectively. Chlorogenic acid is the main phenolic compound in guayusa leaves; its concentrations were corroborated by García-Ruiz et al. [6], who have reported 24.10 mg/g DW, while Villacis-Chiriboga et al. [5] have reported a value of 7.63 mg/g DW for young leaves and 6.51 mg/g DW for old leaves. The caffeine content in guayusa leaves has been widely studied, with values ranging from 29 to 32 mg/g DW [24] to 36 mg/mL [25], and lower concentrations reported by Luna-Fox et al. [26] (15.2 mg/g DW) and Wise et al. [27] (19.08 mg/g).

Supercritical CO_2_ extraction techniques have been considered as ecological or green procedures for processing natural sources due to the preservation of bioactive properties during the extraction and the reduced use of organic solvents. Supercritical processing of natural matter is an alternative to classic technologies and provide special characteristics to the obtained product, such as micronization, purification, and enhanced solubility [18,28,29].

The SAE technique showed precipitation yields between 7.06% and 24.54%; these yields were significantly lower than those reported by Villanueva et al. [14] for the SAE processing of green tea leaves (27.6% to 64%), and by Meneses et al. [13] for mango by-products (63.7% to 74.3%). The modification of pressure and temperature in SAE allows for the tuning of the CO_2_ density; as a rule, at a higher density, the SC-CO_2_ increases its extraction capacity. In this study, the higher precipitation yield (24.54%) was achieved at 120 bar and 35 °C, corresponding to a CO_2_ density of 766.7 kg/m^3^, which is in line with observations by Martín et al. [30], who found that higher CO_2_ densities generally led to improvement extraction capacities, such as for the fractionation of Ryanodol from *Persea indica*, with precipitation yields between 19.4% and 56.1%, where the highest yield corresponded to the higher density (815.29 kg/m^3^).

The measurement of CGA and caffeine content in the SAE precipitate and fractionate allowed us to highlight and verify the concentrations of antioxidants and the fractionation of caffeine. The highest CGA concentration observed was 237.86 ± 13.83 mg CGA/g precipitate, which resulted in a concentration factor of 5.53 relative to the initial ethanolic extract (43.02 ± 5.23 mg CGA/g extract), with nearly 100% recovery of CGA. While the concentration of caffeine was reduced to 0.14 mg caffeine/g precipitate, which corresponded to a factor of concentration of 0.002, indicating the efficient removal of caffeine from the precipitate fraction. The caffeine was predominantly recovered in the fractionate fraction, with the highest concentration of 116.78 ± 4.33 mg caffeine/g precipitate at 100 bar and 35 °C, corresponding to a concentration factor of 1.48. This effective fractionation process was comparable to that reported by Villanueva et al. [14], who successfully enriched epigallocatechin gallate (EGCG) from green tea extracts while removing caffeine using SAE, with a reduction in caffeine (93%) for ethyl lactate extract and a precipitation yield of 54%. Their findings also suggest that increasing the pressure and temperature improves the separation of caffeine from antioxidant compounds, which aligns with the results of this study. The SAE process produced a precipitate with less than 1% caffeine, which could be classified as decaffeinated based on European standards [31]. This outcome holds potential for developing high-antioxidant, decaffeinated guayusa products, as previously suggested by Villanueva et al. [14] for EGCG-enriched precipitates with reduced caffeine content.

In terms of the antioxidant capacity, the SAE precipitates showed significant enhancement compared with the initial ethanolic extract. The highest TPC value was 561.52 ± 15.71 mg GAE/g extract at 120 bar and 45 °C, and the corresponding ABTS and DPPH values were 196.96 ± 16.5 mg GAE/g precipitate and 849.36 ± 0.86 mg GAE/g precipitate, respectively. For all cases, the antioxidant capacities of the precipitates were increased considerably due to the concentration of antioxidant compounds, particularly CGA in the SAE precipitate. Villanueva et al. [14] presented high contents of phenolic compounds with an increase of around 12–25% of the initial extract, possibly due to the concentration of antioxidants (EGCG). However, this contrasts with Meneses et al. [13], who observed a minimal variation in the antioxidant activity during SAE processing of mango by-products; these differences could have been due to the use of a highly purified ethanolic extract, which may have limited the further concentration of antioxidants.

Regarding the co-precipitation, it was observed that the presence of PVP did not considerably alter the ability of the SAE process to fractionate caffeine and concentrate antioxidants in the co-precipitate. Additionally, spherical microparticles were obtained that could indicate the effective encapsulation of bioactive compounds. Montes et al. [17] observed similar results when using PVP in the SAS process to produce microparticles with antioxidant activity from moringa leaves, where PVP was utilized as an inducer in the precipitation. Similar to our results, the presence of PVP increased the precipitation yield of microparticles, suggesting that PVP played a critical role in improving the process efficiency. In addition, the IC_50_ of the SAE precipitate was 0.04 ± 0 mg/mL for DPPH and 0.18 ± 0.01 mg/mL for ABTS, which corresponded to the high concentration of antioxidants (CGA) achieved through the SAE process. Guamán-Balcázar et al. [32] have also reported an increase in the antioxidant activity of mango leave precipitates processed using SAE compared with the initial extract. The IC_50_ values reported for guayusa leaves show significant variation based on extraction methods. Cadena-Carrera et al. [23] presented an IC_50_ of 0.15 mg/mL for Soxhlet extraction with ethanol, 4.42 mg/mL for SC-CO_2_ extraction, and 1.37 mg/mL for SC-CO_2_ with ethanol as the cosolvent. These differences were attributed to variations in the polarity and the types of compounds extracted. In the case of co-precipitates, the presence of PVP reduced the antioxidant capacity, as indicated by the increase in the IC_50_ values with higher PVP ratios (Table 6). Similar results were observed by Montes et al. [17], who reported an IC_50_ of 15.38 µg/mL for the ethanol extract from moringa leaves, which decreased in the precipitates due to the presence of the encapsulant.

The particle sizes were determined for treatments 1, 2, 6, and 7; in all cases, the particles obtained were nanometric. As previously reported by Baldino et al. [33], smaller particle sizes are typically associated with a higher CO_2_ density in the SAE process. Guamán-Balcázar et al. [16] also demonstrated that the incorporation of PVP in the co-precipitation of mango leaf ethanolic extracts favored the formation of spherical particles compared with the precipitates without PVP. A similar trend was observed in this study with guayusa extracts without PVP (Figure 1a), which exhibited slightly agglomerated spherical microparticles with a wide range of PSD, as shown in Figure 2, while those formed in the presence of PVP (Figure 1b) displayed spherical particles with a narrower PSD (Figure 2). The coprecipitation of regular microspheres suggests efficient encapsulation, which is beneficial for the stability and bioavailability of bioactive compounds.

Co-precipitation with PVP has several advantages in terms of the stability and delivery of bioactive compounds. Ozkan et al. [34] reported that SAS co-precipitation enhances the stability, bioaccessibility, and bioavailability of bioactives. Franco and De Marco [12] remarked on the versatility of SAE as a technique in pharmaceutical applications, particularly in modulating drug release by selecting a carrier based on a required therapy, such as PVP. The co-precipitation of antioxidants from guayusa extracts with PVP increases the solubility of poorly soluble compounds.

Furthermore, regarding the potential to produce a decaffeinated antioxidant product using this co-precipitation process, Zhang et al. [20] reported the possibility to scale up the sustainability production of SAE functional ingredients with targeted bioactive profiles. A life cycle assessment could be employed to assess the environmental impact of large-scale implementation, as described by De Marco [11] regarding the supercritical antisolvent precipitation of a PVP/prednisolone system.

## 4. Materials and Methods

### 4.1. Material

*Ilex guayusa* leaves were collected in Gualaquiza (78°32′28.54″ W, 3°25′32.17″ S, and 890 m.a.s.l.) located in the Amazonian province of Morona Santiago, Ecuador. After the collection, the samples were transported immediately to the laboratories of the Chemistry Department of UTPL in Loja. The transportation time was almost 12 h at room temperature (21 °C). The vegetal material was selected according to its color, then washed and disinfected with a sanitization solution (sodium hypochlorite) to eliminate contaminants. After this, the humidity of the leaves was reduced in a convective dryer operating at 40 °C until the humidity was less than 10% weight. The dehydrated material was powdered with an ultracentrifuge mill (Retsch, ZM200, Haan, Germany) to reach a particle size lower than 250 µm. The powdered material was packed in plastic bags and kept protected from light at −4 °C until its use.

### 4.2. Total Extract Preparation

A liquid extract was obtained by dynamic solid–liquid extraction using ethanol (99.5%) as a solvent. The guayusa leaves were put in contact with ethanol in a 1/20 (weight/volume) relationship and the extraction occurred while maintaining the temperature at 45 °C and continuously stirring at 160 rpm for 6 hours. After separating the liquid extract, the solid material was extracted once again under similar conditions. The two liquid extracts were mixed, and the concentration of the extracted material was adjusted to 30 mg/mL through the evaporation of the solvent at 160 mbar and 45 °C.

### 4.3. Supercritical CO_2_ Antisolvent Extraction

**Figure 3:**
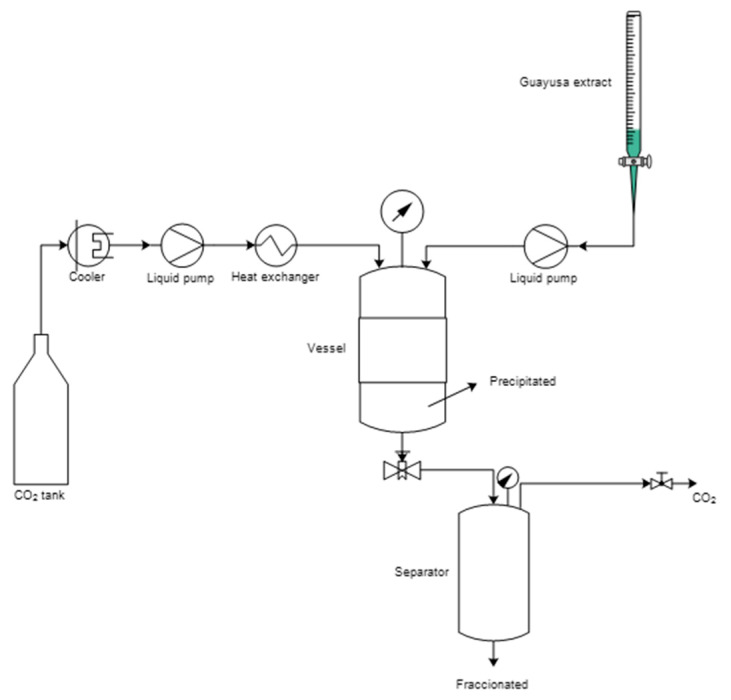
Schematic representation of SC-CO_2_ SAE pilot plant.

The SAE technique was applied to the total ethanolic extract; for this purpose, a homemade supercritical pilot plant was used [35]. The equipment consisted of a precipitation vessel, in which the contact of the continuous flow of ethanolic extract and CO_2_ at supercritical conditions (SC-CO_2_) occurred. Figure 3 shows a schematic representation of the pilot plant. The antisolvent capacity of the SC-CO_2_ was modulated by varying the pressure and temperature from 8 MPa to 12 MPa and 35 °C to 45 °C, respectively.

The SAE technique produces two fractions, a powdered precipitated that was collected inside the precipitation vessel, which was retained by a microporous filter (porosity 1 µm), and a liquid fraction collected in a separator vessel that operated at lower pressure (35 bar); afterward, both fractions were analyzed.

The recovery yield (Equation (1)) was calculated considering the relationship between the total solid contained in each fraction (precipitate and liquid fraction) and the total solid in the inlet extract.

(1) $$Recovery\;yield\;\left(\%\right)=\left[\frac{total\;solids\;in\;SAE\;fraction}{total\;solids\;in\;initial\;extract}\right]\times 100$$

### 4.4. Evaluation of Antioxidant Capacity

The antioxidant capacity was measured by two colorimetric methods, DPPH• and ABTS•+, according to Martinez et al. [36]. The sample to be analyzed was diluted at a concentration of 1 mg/mL. A total of 150 µL was mixed separately with 2850 µL of a DPPH solution and ABTS solution (Sigma-Aldrich, Steinheim, Germany), and after the reaction time, the variation in the absorbance was recorded. The DPPH and ABTS solutions were standardized at an absorbance of 1.1 ± 0.02 measured at 515 nm and 734 nm wavelengths, respectively, using a Cary 60 UV–Vis spectrometer (Agilent Technologies, Santa Clara, CA, USA). In both cases, Trolox (Sigma-Aldrich, Steinheim, Germany) was used as the standard, obtaining the following calibration equations:

DPPH: *y* = −0.0011 × *x* + 0.9803 (2)

ABTS: *y* = −0.0028 × *x* + 1.0035 (3)

where *x* is the concentration of the Trolox solutions in µMol TE/L and *y* is the absorbance, where both had an R^2^ higher than 0.99. The results are expressed as micromoles of Trolox equivalent by gram of extract (µMol TE/g extract).

### 4.5. Determination of Total Phenols 

The Folin–Ciocalteu colorimetric method was used to measure the total phenol content, as mentioned by Martinez et al. [36]. Briefly, 150 µL of sample was mixed with 2400 µL of distillate water and 150 µL of a 0.25 N Folin–Ciocalteu solution (Sigma-Aldrich, Steinheim, Germany); this mixture was allowed to react for 3 min. Then, 300 µL of 1 N sodium carbonate was added and a final reaction occurred over 2 h. The variation in absorbance was recorded at a 725 nm wavelength. Gallic acid (Sigma-Aldrich, Steinheim, Germany) was used as the standard, and the calibration equation had an R^2^ higher than 0.99.

TPC: *y* = 0.0021 × *x*+ 0.0077 (4)

where *x* is the concentration of the Gallic acid solution in mg/mL and *y* is the absorbance. The results are expressed as milligrams of Gallic Acid equivalent by gram of extract (mg GAE/g extract).

### 4.6. Identification of Chemical Compounds

The identification of chemical components was performed in an HPLC-DAD Ultimate 3000 (Thermo Scientific LTQ XL, Germering, Germany) chromatograph equipped with a photodiode array detector. The chromatographic method reported by Prencipe et al. [37] was slightly modified for the quantification. The mobile phase consisted of the mixture of two solvents: (A) aqueous solution of formic acid (0.1%) and (B) pure acetonitrile. The linear gradient of B was 0–15 min, 20%; 15–25 min, 30%; 25–45 min, 50%; and 45–50 min, 50%, at a 1 mL/min total flow.

An injection volume of 5 µL was used. The compounds were separated in a C18 column (25 cm × 0.46 cm DI, 5 µm particle size, Supelco, Inc., Bellefonte, PA, USA) with an oven temperature of 25 °C. The phenolic compounds were identified by comparison of the retention time with those obtained with pure standards: chlorogenic acid, caffeine, and rutin (Sigma-Aldrich, Steinheim, Germany). The UV–Visible spectra were acquired at 330 nm for hydroxycynnamic acids and 360 nm for flavonols. The results are expressed as milligrams of phenolic compound by gram of extract (mg/g extract).

### 4.7. Supercritical Antisolvent Co-Precipitation

The use of polyvinylpyrrolidone (PVP MW 10000) (Sigma-Aldrich, Steinheim, Germany) as the encapsulation material was proposed with the aim of protecting the antioxidant properties of guayusa extract and to improve the precipitation yield. For this purpose, PVP was added to the ethanolic extract (30 mg/mL) in two proportions: 1:1 and 1:2 (total solids in extract–PVP). The supercritical antisolvent procedure was similar to that described in Section 2.3; 30 mL of extract–PVP solution was used, and the operating supercritical pressure and temperature were selected considering the SAE results. The precipitated fraction was characterized by the particle size and chemical composition.

### 4.8. Radical Scavenging Activity and IC_50_ in DPPH and ABTS Assays

The antioxidant activities of the co-precipitates were measured by the analysis of the free radical scavenging capacity using the DPPH and ABTS radicals, following the method described by Meneses et al. [13]. Different concentrations of co-precipitate, ranging from 0.05 to 1 mg/mL in ethanol, were analyzed. The radical scavenging activity was calculated using Equation (5):

(5) $$Radical\;scavenging\;\left(\%\right)=\left[\frac{A_{S}-A_{CP}}{A_{S}}\right]\times 100$$

where *A_S_* is the absorbance of the initial DPPH or ABTS solution and *A_CP_* is the absorbance of the co-precipitates. The inhibition concentration (IC_50_), which represents the concentration of co-precipitates required to reduce the initial DPPH or ABTS concentration by 50%, was derived from the respective plots for all co-precipitates, and the results are expressed in mg of co-precipitate per mL (mg CP/mL).

### 4.9. Particle Size Distribution

SEM images of the co-precipitates were acquired using a scanning electron microscope (FEG-SEM) (TESCAN, Mira 3, Brno, Czech Republic). In this assay, the precipitated samples were dispersed over an adhesive carbon tab and coated with gold film using a sputter coater (Quorum Technologies mod. Q105R, Lewes, UK). The particle size and particle distribution were calculated from SEM images using the ImageJ/FIJI image analysis software (version 1.46, Wayne Rasband, NIH, USA). For each PSD, approximately 230 particles were analyzed and processed using Microcal Origin Software (version 8.0 Origin Lab Corporation, Northampton, MA, USA).

### 4.10. Statistical Analysis

The procedures of characterization of the ethanolic extract and SAE processing were performed in triplicate. The data were collected in a Microsoft Excel (Microsoft 365) sheet. In the SAE treatments, a 3^2^ central composite experimental design was applied to evaluate the effect of the supercritical CO_2_ pressure and temperature on the responses of the precipitated fraction: recovery yield, total phenols content, antioxidant capacity, and chlorogenic acid concentration. The significative effect of the factors was determined by an ANOVA (*p* < 0.05) with 95% confidence. For the co-precipitate treatments, an ANOVA and Fisher’s mean differences test were performed to identify the effects of the extract–PVP ratio, the supercritical pressure and temperature over the responses of co-precipitates: recovey yield, total phenols content, chlorogenic acid concentration and IC_50_. The software used for the statistical analysis was Minitab version 19.1 (Minitab, LLC, State College, PA, USA). The values are expressed as the mean value ± standard deviation.

## 5. Conclusions

*Ilex guayusa* L. leaves were valorized through the production of encapsulated high-antioxidant content. The SAE process demonstrated its efficacy in selectively concentrating chlorogenic acid and removing caffeine from guayusa leaf extracts to values less than 1% by weight; in this way, it was possible to produce a decaffeinated antioxidant-rich functional ingredient. The SAE recovery yield showed values that depended on the operative conditions and the initial content of solids of guayusa extracts. The SAE process significantly enhanced the antioxidant capacity of the precipitates for DPPH and ABTS assays. In the SAE optimal conditions, 120 bar and 35 °C, the SAE precipitate showed values of 218.63 ± 15.49 µMol TE/g and 764.52 ± 53.57 µMol TE/g for ABTS and DPPH, respectively, while chlorogenic acid was presented as 213.76 ± 22.39 mg/g precipitate. The co-precipitation of guayusa extracts with PVP through SAE not only improved the recovery yield from 24.54% to 71.53% but also retained the antioxidant purification and caffeine fractionates effectively, where the encapsulation reached with SAE co-precipitation enhanced the stability and bioavailability of the bioactive compounds. The formation of uniform, spherical nanoparticles suggests successful encapsulation, making this approach promising for the development of functional food or pharmaceutical ingredients. These results suggest that guayusa leaves can be effectively valorized to produce high-value products with concentrated antioxidant content and low caffeine levels.

## Figures and Tables

**Table 1:** Characterization of ethanolic extract of *Ilex guayusa* L.

	*Ilex guayusa* L. Ethanolic Extract	
	Mean	SD
Extraction yield, % (g extract/g dry leaves)	33.61	2.27
Total phenols content, mg GAE/g extract	78.42	1.38
DPPH, µMol Trolox/g extract	75.6	12.1
ABTS, µMol Trolox/g extract	59.8	5.72
Phenolic compounds, mg/g extract		
Chlorogenic acid	43.02	5.23
Caffeic acid	1.56	0.03
Rutin	2.91	0.01
Caffeine content, mg/g extract	79.16	3.56

GAE: gallic acid equivalent; ABTS: 2,2′-azinobis(3-ethylbenzothiazoline-6-sulfonic acid); DPPH: 2,2-diphenyl-1-picrylhydrazyl; TE: Trolox equivalent.

**Table 2:** Recovery yield in SAE treatments.

Treatment	*p*	T	Recovery Yield *	
	(bar)	(°C)	(%, *w*/*w*)	
			Precipitated	Fractionated
1	80	35	7.06 ± 3.73	69.10 ± 19.56
2	100	35	15.18 ± 0.35	44.19 ± 4.15
3	120	35	24.54 ± 0.40	34.24 ± 2.29
4	80	40	8.85 ± 2.73	73.27 ± 23.64
5	100	40	20.95 ± 4.62	57.71 ± 2.80
6	120	40	17.01 ± 5.49	45.01 ± 0.29
7	80	45	16.00 ± 2.31	61.10 ± 19.49
8	100	45	9.68 ± 2.51	51.14 ± 11.22
9	120	45	10.79 ± 0.09	68.57 ± 29.41

*: values are expressed as mean value and standard deviation.

**Table 3:** Total phenols content in SAE treatments.

Treatment	P	T	Total Phenols Content	
	(bar)	(°C)	(mg GAE/g Extract)	
			Precipitated	Fractionated
1	80	35	425.02 ± 118.08	14.19 ± 2.13
2	100	35	434.54 ± 5.84	9.27 ± 3.93
3	120	35	461.52 ± 10.77	18.32 ± 11.34
4	80	40	449.78 ± 75.87	28.87 ± 17.51
5	100	40	417.08 ± 39.51	16.81 ± 1.12
6	120	40	452.95 ± 23.79	14.35 ± 5.28
7	80	45	510.73 ± 9.43	29.90 ± 22.56
8	100	45	488.19 ± 32.32	8.00 ± 0.34
9	120	45	561.52 ± 15.71	24.35 ± 6.17

GAE: gallic acid equivalent.

**Table 4:** Antioxidant capacity of the products from the SAE treatments measured by ABTS and DPPH assays.

Treatment	P	T	ABTS		DPPH	
	(bar)	(°C)	(µMol TE/g Fraction)		(µMol TE/g Fraction)	
			Precipitate	Fractionate	Precipitate	Fractionate
1	80	35	203.81 ± 66.08	29.80 ± 1.57	653.76 ± 271.06	22.97 ± 3.76
2	100	35	199.58 ± 5.39	29.17 ± 0.79	688.00 ± 15.21	21.14 ± 1.29
3	120	35	218.63 ± 15.49	27.02 ± 0.90	764.52 ± 53.57	31.70 ± 15.66
4	80	40	211.07 ± 45.37	56.87 ± 12.68	688.91 ± 223.92	38.29 ± 26.10
5	100	40	186.31 ± 11.87	38.21 ± 4.94	601.33 ± 44.78	27.06 ± 1.57
6	120	40	200.24 ± 0.42	31.75 ± 2.08	717.55 ± 5.57	24.52 ± 2.58
7	80	45	194.76 ± 14.73	56.63 ± 11.00	796.64 ± 59.14	41.10 ± 29.86
8	100	45	185.95 ± 7.49	21.19 ± 2.41	741.94 ± 116.35	19.83 ± 1.35
9	120	45	196.96 ± 16.50	47.46 ± 7.01	849.36 ± 0.86	36.70 ± 8.70

ABTS: 2,2′-azinobis(3-ethylbenzothiazoline-6-sulfonic acid); DPPH: 2,2-diphenyl-1-picrylhydrazyl; TE: Trolox equivalent.

**Table 5:** Chemical composition of the SAE fractions.

Treatment	P	T	Chlorogenic Acid		Caffeine	
	(bar)	(°C)	(mg/g Fraction)		(mg/g Fraction)	
			Precipitate	Fractionate	Precipitate	Fractionate
1	80	35	144.36 ± 1.05	14.51 ± 4.52	0.00 ± 0.00	70.96 ± 4.25
2	100	35	118.30 ± 8.24	4.05 ± 0.03	0.00 ± 0.00	116.78 ± 4.33
3	120	35	213.76 ± 22.39	9.68 ± 2.44	0.00 ± 0.00	91.17 ± 10.84
4	80	40	178.37 ± 4.70	12.01 ± 0.01	0.00 ± 0.00	80.99 ± 0.03
5	100	40	154.85 ± 44.35	5.58 ± 1.02	0.14 ± 0.198	101.19 ± 9.49
6	120	40	152.69 ± 35.68	4.98 ± 0.98	0.00 ± 0.00	87.43 ± 16.62
7	80	45	155.26 ± 6.65	5.07 ± 1.22	0.00 ± 0.00	75.73 ± 0.75
8	100	45	195.97 ± 45.12	8.15 ± 4.59	0.00 ± 0.00	73.46 ± 7.86

**Table 6:** Results for SAE co-precipitation treatments.

Treatment *	T	P	Ext–PVP	Yield	TPC	CGA	Caffeine
	(°C)	(Bar)	Ratio	(%)	(mg GAE/g CP)	(mg/g CP)	(mg/g CP)
1	35	120	0	24.54 ± 0.40 ^a^	465.61 ± 31.04 ^a^	30.11 ± 4.27 ^a^	1.76 ± 0.18 ^a^
2	35	120	1:01	65.83 ± 2.95 ^b,c^	348.10 ± 34.49 ^b,c^	7.15 ± 0.22 ^b^	0.61 ± 0.05 ^b^
3	35	120	1:02	66.67 ± 3.93 ^b,c^	240.78 ± 13.80 ^e^	5.14 ± 0.39 ^b,c^	0.14 ± 0.01 ^d,e^
4	35	150	1:01	67.29 ± 2.65 ^b,c^	310.05 ± 1.38 ^c,d^	5.33 ± 0.53 ^b,c^	0.46 ± 0.05 ^b,c^
5	35	150	1:02	71.53 ± 2.36 ^b^	255.41 ± 8.28 ^e^	2.65 ± 0.18 ^c^	0.13 ± 0.06 ^d,e^
6	45	120	1:01	62.29 ± 2.06 ^c^	370.54 ± 15.18 ^b^	6.47 ± 0.07 ^b,c^	0.30 ± 0.02 ^c,d^
7	45	120	1:02	71.11 ± 4.52 ^b^	277.85 ± 16.56 ^d,e^	3.71 ± 0.16 ^b,c^	0.07 ± 0.01 ^e^

PVP: polyvinylpyrrolidone; TPC: total phenols content; CGA: chlorogenic acid; GAE: gallic acid equivalent; CP: co-precipitate. *: treatments performed with a new ethanolic extract adjusted at 30 mg/mL in solids concentration; ^a–e^: mean values with different letters in the same column indicate statistically significant differences (*p* < 0.05) among treatments for the tested assays.

**Table 7:** IC_50_ of SAE co-precipitates for DPPH and ABTS assays.

Treatment	T	P	Ext–PVP	DPPH	ABTS
	(°C)	(bar)	Ratio	(IC_50_, mg/mL)	(IC_50_, mg/mL)
1	35	120	0	0.04 ± 0.00 ^a^	0.18 ± 0.01 ^a^
2	35	120	1:01	0.51 ± 0.01 ^b^	0.77 ± 0.02 ^b^
3	35	120	1:02	0.94 ± 0.08 ^c,d^	1.68 ±0.04 ^c^
4	35	150	1:01	0.75 ± 0.07 ^e^	1.0 ± 0.00 ^d^
5	35	150	1:02	1.0 ± 0.00 ^c^	2.09 ± 0.02 ^e^
6	45	120	1:01	0.54 ± 0.04 ^b^	0.74 ± 0.01 ^b^
7	45	120	1:02	0.86 ± 0.01 ^d^	2.18 ± 0.03 ^f^

^a–f^: Mean values with different letters in the same column indicate statistically significant differences (*p* < 0.05) among the treatments for the tested assays.

## Data Availability

The original contributions presented in this study are included in this article; further inquiries can be directed to the corresponding author.
